# Supplementary material for: Patch formation driven by stochastic effects of interaction between viruses and defective interfering particles
Source: PLoS Comput Biol. 2023 Oct 2;19(10):e1011513. doi: 10.1371/journal.pcbi.1011513 (PMC10569632; doi:10.1371/journal.pcbi.1011513)
Supplement: S1 Appendix — (PDF) [file pcbi.1011513.s001.pdf]

Appendix

Virus infection without DIPs

Setting $D = C_D = C_{VD} = C^*_{VD} = 0$ in Eqs (1) and Eqs (2) gives the system

$$\begin{aligned}
\frac{\partial V}{\partial t} &= d_V \nabla^2 V + \alpha_1 C^*_V - \delta_V V, \\
\frac{\partial C}{\partial t} &= \alpha_C C(1 - C_T/K) - \gamma_1 CV - \delta_C C, \\
\frac{\partial C_V}{\partial t} &= \gamma_1 CV - \nu_1 C_V - \delta_{CV} C_V, \\
\frac{\partial C^*_V}{\partial t} &= \nu_1 C_V - \beta_1 C^*_V.
\end{aligned} \tag{A.1}$$

To study the dynamics of Eqs (A.1), we obtain the following result for the homogeneous steady states of the system. Roughly, the number of steady states decreases from 3 to 1 as δ_C/α_C increases pass two critical values.

Lemma .1. *Let* $Z = \frac{\delta_V \beta_1(\delta_{CV}+\nu_1)}{\gamma_1\alpha_1\nu_1}$. *For studying the non-negative homogeneous steady states of Eqs (A.1), there are three cases:*

(1) If $\alpha_C \leq \delta_C$, *there is only one steady state* $E_0 = (0, 0, 0, 0)$.

(2) If $\alpha_C \left(1 - \frac{Z}{K}\right) \leq \delta_C < \alpha_C$, *there are two steady states,* E_0 *and* $E_1 = (0, \frac{K(\alpha_C - \delta_C)}{\alpha_C}, 0, 0)$.

(3) If $\delta_C < \alpha_C \left(1 - \frac{Z}{K}\right) < \alpha_C$, *there are three steady states,* E_0, E_1 *and* $E_2 = (\overline{V}, Z, \overline{C}_V, \overline{C}^*_V)$ *where*

$$\begin{aligned}
\overline{C}_V &= \frac{Z\left(\alpha_C\left(1 - \frac{Z}{K}\right) - \delta_C\right)}{\nu_1 + \delta_{CV} + (\beta_1 + \nu_1)\alpha_C Z/(\beta_1 K)}, \\
\overline{C}^*_V &= \frac{\nu_1}{\beta_1}\overline{C}_V, \\
\overline{V} &= \frac{\alpha_1\nu_1}{\delta_V\beta_1}\overline{C}_V.
\end{aligned}$$

Proof. First, we consider the homogeneous steady state equations of Eqs (A.1),

$$\begin{aligned}
0 &= \alpha_1 x_4 - \delta_V x_1, \\
0 &= \alpha_C x_2(1 - (x_2 + x_3 + x_4)/K) - \gamma_1 x_2 x_1 - \delta_c x_2, \\
0 &= \gamma_1 x_2 x_1 - \nu_1 x_3 - \delta_{CV} x_3, \\
0 &= \nu_1 x_3 - \beta_1 x_4.
\end{aligned} \tag{A.2}$$

By the first and the last equations above, we obtain $x_1 = \alpha_1\nu_1 x_3/(\delta_V\beta_1)$ and $x_4 = \nu_1 x_3/\beta_1$. Substitute $x_1 = \alpha_1\nu_1 x_3/(\delta_V\beta_1)$ to the third equation, we have

$$0 = \gamma_1\alpha_1\nu_1 x_2 x_3/(\delta_V\beta_1) - \nu_1 x_3 - \delta_{CV} x_3,$$

$$0 = \gamma_1\alpha_1\nu_1(x_2 - Z)x_3/(\delta_V\beta_1).$$

$$x_3 = 0 \text{ or } x_2 = Z.$$

If $x_3 = 0$, we obtain that $x_1 = x_4 = 0$. Consider the second equation with $x_1 = x_3 = x_4 = 0$, we have

$$0 = (\alpha_C - \delta_C - \alpha_C x_2/K)x_2,$$

which leads to two possible non-negative solution $x_2 = 0$ or $x_2 = K(\alpha_C - \delta_C)/\alpha_C$ if $\alpha_C > \delta_C$.

If $x_2 = Z$, we consider the addition of the second and the third equations with $x_1 = \alpha_1\nu_1 x_3/(\delta_V\beta_1)$ and $x_4 = \nu_1 x_3/\beta_1$. If $\delta_C < \alpha_C\left(1 - \frac{Z}{K}\right)$, we can find a positive solution for x_3,

$$x_3 = \frac{Z\left(\alpha_C\left(1 - \frac{Z}{K}\right) - \delta_C\right)}{\nu_1 + \delta_{CV} + (\beta + \nu_1)\alpha_C Z/(\beta_1 K)}.$$

We proved the existence of the homogeneous steady states for the three cases. □

Proposition .2. *If $\alpha_C < \delta_C$, the solution will approach to $E_0 = (0, 0, 0, 0)$ as $t \to \infty$.*

Proof. By considering the second equations in Eqs (A.1),

$$\frac{\partial C}{\partial t} = \alpha_C C(1 - C_T/K) - \gamma_1 CV - \delta_C C \leq \alpha_C C - \delta_C C = (\alpha_C - \delta_C)C,$$

which leads to

$$C(t, \vec{x}) \leq C(0, \vec{x})e^{(\alpha_C - \delta_C)t}.$$

If $\alpha_C - \delta_C < 0$, then $C(t, \vec{x})$ will approach to zero as $t \to \infty$.

Through considering the equations for C_V, C_V^* and V one by one, it is easy to show that when $C(t, \vec{x})$ approaches zero, C_V, C_V^* and V all approach zero as $t \to \infty$. □

Proposition .3. *If $\alpha_C \left(1 - \frac{Z}{K}\right) < \delta_C < \alpha_C$ and $C(0, \vec{x}) > 0$, the solution will approach to $E_1 = (0, \frac{K(\alpha_C - \delta_C)}{\alpha_C}, 0, 0)$ as $t \to \infty$.*

Proof. Let $f(y_1, y_2) = (\alpha_C(1 - y_1/K) - \gamma_1 y_2 - \delta_C)$. By $\alpha_C \left(1 - \frac{Z}{K}\right) < \delta_C$, we observe that $f(y_1, y_2) < 0$ for any $y_1 > Z$ and $y_2 > 0$. If $C \geq Z$,

$$\frac{\partial C}{\partial t} = f(C_T, V)C \leq f(C, 0)C < 0.$$

It implies that $C(t, \vec{x}) < Z$ as $t \to \infty$.

Define $Y = (\nu_1 + \delta_{CV})(\alpha_1 C_V^* + \beta_1 V) + \alpha_1 \nu_1 C_V$. Consider the derivative of Y with respect to t,

$$\frac{\partial Y}{\partial t} = \beta_1(\nu_1 + \delta_{CV})d_V \nabla^2 V + (C - Z)V\alpha_1\nu_1\gamma_1.$$

Take integration over the spatial domain Ω, we can obtain

$$\frac{\partial \int_\Omega Y dA}{\partial t} = \int_\Omega (C - Z)V\alpha_1\nu_1\gamma_1 dA.$$

If V is not zero, the right-hand side becomes negative as $t \to \infty$, then Y will decrease. If there exists $T > 0$ such that V becomes zero for $t > T$, it is easy to show that C_V and C_V^* approach to zero; if not, Y will approach to zero and It implies that V, C_V and C_V^* approach to zero as $t \to \infty$. When $t \to \infty$, V, C_V and C_V^* approach to zero and

$$\frac{\partial C}{\partial t} = \alpha_C C(1 - C/K) - \delta_C C$$

If C is not zero with the condition $\alpha_C \left(1 - \frac{Z}{K}\right) < \delta_C < \alpha_C$, the equation above implies that $C \to \frac{K(\alpha_C - \delta_C)}{\alpha_C}$ as $t \to \infty$.

□

Our set of parameters does not meet the conditions of the propositions above. Therefore it can guarantee that species in the system will not become extinct in the following simulations.
